# Immunohistochemical characteristics and potential therapeutic regimens of hepatoid adenocarcinoma of the stomach: a study of 139 cases

**DOI:** 10.1002/cjp2.343

**Published:** 2023-11-16

**Authors:** Xuesong Yang, Yan Wu, Anqiang Wang, Xiuli Ma, Kai Zhou, Ke Ji, Xin Ji, Ji Zhang, Xiaojiang Wu, ZhongWu Li, Zhaode Bu

**Affiliations:** ^1^ Key Laboratory of Carcinogenesis and Translational Research (Ministry of Education/Beijing), Center of Gastrointestinal Cancer Peking University Cancer Hospital & Institute Beijing PR China; ^2^ Key Laboratory of Carcinogenesis and Translational Research (Ministry of Education/Beijing), Department of Pathology Peking University Cancer Hospital & Institute Beijing PR China

**Keywords:** hepatoid adenocarcinoma of stomach, immunohistochemistry, targeted therapy

## Abstract

Hepatoid adenocarcinoma of stomach (HAS) is a special subtype of gastric cancer with poor prognosis. Immunohistochemical analysis could provide important clues for the treatment of HAS. A total of 159 patients were diagnosed as HAS and 139 were enrolled in this study. Statistical differences were determined using relative test methods and survival analyses were performed by the Kaplan–Meier method to find survival differences. All tumors in this study were negative for Epstein–Barr virus‐encoded small RNAs (EBERs) and almost all showed no loss of mismatch repair (MMR) proteins and were positive for alpha fetoprotein (AFP or spalt like transcription factor 4 (SALL4). About half of the tumors had a positive programmed death‐ligand 1 combined positive score (CPS) and 17.3% were positive for human epidermal growth factor receptor 2 (HER2). In addition, there was a relatively high proportion of cmet expression. We also found that HAS patients with recurrent disease treated by emerging therapy had a better survival than those treated with traditional chemotherapy (*p* = 0.002, median recurrence‐to‐death survival: 23 months versus 6 months); HAS patients who received anti‐HER2 therapy or harbored MMR deficiency had favorable prognosis. Overall, high proportions of MMR protein proficiency, positivity for AFP or SALL4, overexpression of HER2, CPS and cmet, as well as negative EBER findings, are distinctive characteristics of HAS patients. While negative EBER and MMR proficiency indicate molecular features of HAS, positivity for AFP or SALL4 could aid in the diagnosis of HAS. In addition, HAS patients could benefit from anti‐HER2 therapy, immunotherapy, and anti‐angiogenesis therapy.

## Introduction

Hepatoid adenocarcinoma of stomach (HAS) is a rare subtype of gastric cancer, accounting for 0.3–1% of all gastric cancer [1, 2] with an incidence of about 0.58–0.83 cases per million people [3]. Though the incidence is relatively low, HAS has affected the whole treatment outcome of gastric cancer in view of its high malignancy and poor prognosis [3]. Lymph node and liver metastasis are often present at diagnosis, which also results in less favorable therapeutic efficacy [1]. This tumor type has a unique hepatoid appearance of large polygonal eosinophilic neoplastic cells, similar immunohistochemistry (IHC) to hepatocellular carcinoma, and occasionally with elevated serum alpha fetoprotein (AFP) level [4].

The World Health Organization (WHO) criteria for HAS rely on specific morphological features, regardless of serum AFP level [5, 6]. However, some researchers have found that these criteria may cause an underestimation and misdiagnosis of HAS [5, 7]. They also concluded that IHC and serum AFP levels are important to the diagnosis of HAS. There are already several studies regarding how serum AFP can improve the accuracy of diagnosis [8, 9]. However, due to the rarity of HAS, the IHC and Epstein–Barr virus‐encoded small RNA (EBER) features of HAS have not been fully acknowledged [9, 10]. According to previous research, HAS presents distinctive traits such as negativity for EBER, predominance of microsatellite stability (MSS), and high human epidermal growth factor receptor 2 (HER2) positive rate [11, 12]. Nonetheless, the limited sample size affects the reliability of these conclusions.

Anti‐HER2 and anti‐PD‐1 therapy are considered to be promising treatments for HAS [13]. In general, the therapeutic effectiveness of anti‐HER2 and anti‐PD‐1 treatment is closely related to the amplification status of HER2 and combined positive score (CPS) for programmed death‐ligand 1 (PD‐L1) in the field of gastric cancer [14, 15]. In previous studies, the HER2 positive rate determined by IHC ranged from 14.3% to 60% [3, 12, 13, 16, 17]. However, this rate was not usually validated by fluorescence *in situ* hybridization (FISH). The CPS, as reported by Li *et al*, was 1 or more in nearly half of HAS patients [12]. Nonetheless, the exact CPS was not accessible and the relationship between CPS and survival outcome was insufficiently explored in this study.

Recently, HAS has attracted much attention for its specific newly described molecular traits. In 2021, Liu *et al* found that 57.4% (31/54) of HAS patients had *MET* gene amplification [18]. As a proto‐oncogene, *MET* is a member of the RTK family and encodes the high‐affinity receptor for a hepatocyte growth factor receptor (HGFR) [19]. Both *MET* and HGFR are important in the process of cell growth, migration, morphogenic differentiation, and angiogenesis. And the dysfunction of *MET* and HGFR is closely related to the poor prognosis of some human cancers [20]. Fortunately, promising results have been obtained with *MET* inhibitors in the treatment of non‐small cell lung cancer (NSCLC) [21, 22]. Nonetheless, cmet IHC expression status in HAS has not been reported.

Thus, we designed this research to explore the IHC and EBER features of HAS. Some IHC with promising therapeutic potential might benefit HAS patients and provide the basis for subsequent exploration of new treatments.

## Materials and methods

### Patient enrollment

We included patients who were diagnosed with HAS and underwent gastrectomy from October 2009 to July 2021 at the Center of Gastrointestinal Cancer of Peking University Cancer Hospital. Patients with the following conditions were excluded: existing intraperitoneal metastasis (including peritoneal metastasis and positive cytology) before or during the operation; R1 or R2 resection; metastatic gastric tumor; primary malignant tumor of other organs (except low‐grade malignant tumor); and over 80 years old.

### Disease management

All gastric cancer patients in this study had undergone endoscopy and biopsy pathology to confirm the diagnosis of gastric cancer. Abdominal and pelvic computed tomography was performed to determine the clinical stage of the gastric cancer. Laboratory tests and cardiorespiratory function examination were also conducted to assess the tolerance to surgery. After preoperative examination, a multidisciplinary team conference provided treatment recommendations according to the patients' clinical situation and the National Comprehensive Cancer Network (NCCN) guidelines [23]. Gastrectomy was then performed by a surgical team that included at least one veteran surgeon. According to the postoperative pathological results, patients were given/not given postoperative treatment. Postoperative treatment was given mainly for common gastric cancer based on the NCCN guidelines. Chemotherapy, targeted therapy, immunotherapy, and anti‐angiogenesis therapy were the primary postoperative therapeutic regimens. After postoperative treatment, periodic follow‐up was conducted: every 3 months in the first 2 years, every 6 months during years 2–5, and every year thereafter. Follow‐up continued until death or a predefined deadline (17 February 2023).

### Data collection

Detailed clinical data of HAS patients were collected by two independent researchers. Patient ID, gender, age, BMI, history of tumor, whether perioperative therapy (if received, therapeutic regimen and treatment cycle were also recorded), surgical details, and pathological results were acquired from electronic medical records. Some patients had insufficient IHC or FISH tests and these were carried out if the postoperative pathological resection was available. The overall survival (OS) and recurrence‐free survival (RFS) were the primary and secondary endpoints, respectively. Recurrence‐to‐death survival (RDS) was also recorded.

### Definition

The diagnosis of HAS was in accord with the fifth World Health Organization Classification of Digestive System Tumors. WHO advocated that HAS diagnosis mainly relied on the distinctive pathological morphology of large polygonal eosinophilic neoplastic cells (also called hepatoid area), regardless of the serum AFP level. Notably, IHC for AFP was also used to help identify hepatoid areas in some difficult pathological sections. All HAS patients in this study were diagnosed based on the presence of hepatoid areas. OS was defined as the time from receiving the first treatment (neoadjuvant chemotherapy or surgery) to death caused by any reason or the predefined deadline. Similarly, RFS referred to the time until gastric cancer recurrence, metastasis of gastric cancer, or the predefined deadline. In addition, RDS was defined as the time from recurrence to death caused by any reason or the predefined deadline.

### Immunohistochemistry and fluorescence *in situ* hybridization

Representative formalin‐fixed and paraffin‐embedded blocks were cut into 4‐mm‐thick slices. The details of the primary antibodies and IHC platforms are listed in supplementary material, Table S1. All primary antibodies were pre‐diluted by the supplier.

The Ruschoff/Hofmann method was used to score HER2 IHC staining; 0 (negative): no reactivity or membranous reactivity <10% of tumor cells; 1+ (negative): faint/barely perceptible membranous reactivity in ≥10% of tumor cells; 2+ (equivocal): weak‐to‐moderate complete, basolateral, or lateral membranous reactivity in ≥10% of tumor cells; and 3+ (positive): strong, complete, basolateral, or lateral membranous reactivity in ≥10% of tumor cells. Cases with HER2 scores of 2+ were further tested by FISH using the Histra *HER2* FISH Detection Kit (Jokoh, Tokyo, Japan) as per the manufacturer's instructions. For each specimen, the total number of *HER2* and chromosome enumeration probe 17 (CEP17) signals were counted in at least 20 tumor cell nuclei per tissue section, and those with HER2/CEP17 ratios ≥2.0 were defined as *HER2* amplification. EBER FISH was performed using EBER probes (Leica Biosystems, Melbourne, Australia) to detect EBV status. HER2 3+ or amplification was categorized as positive, while any other cases were considered negative.

**Figure 1:**
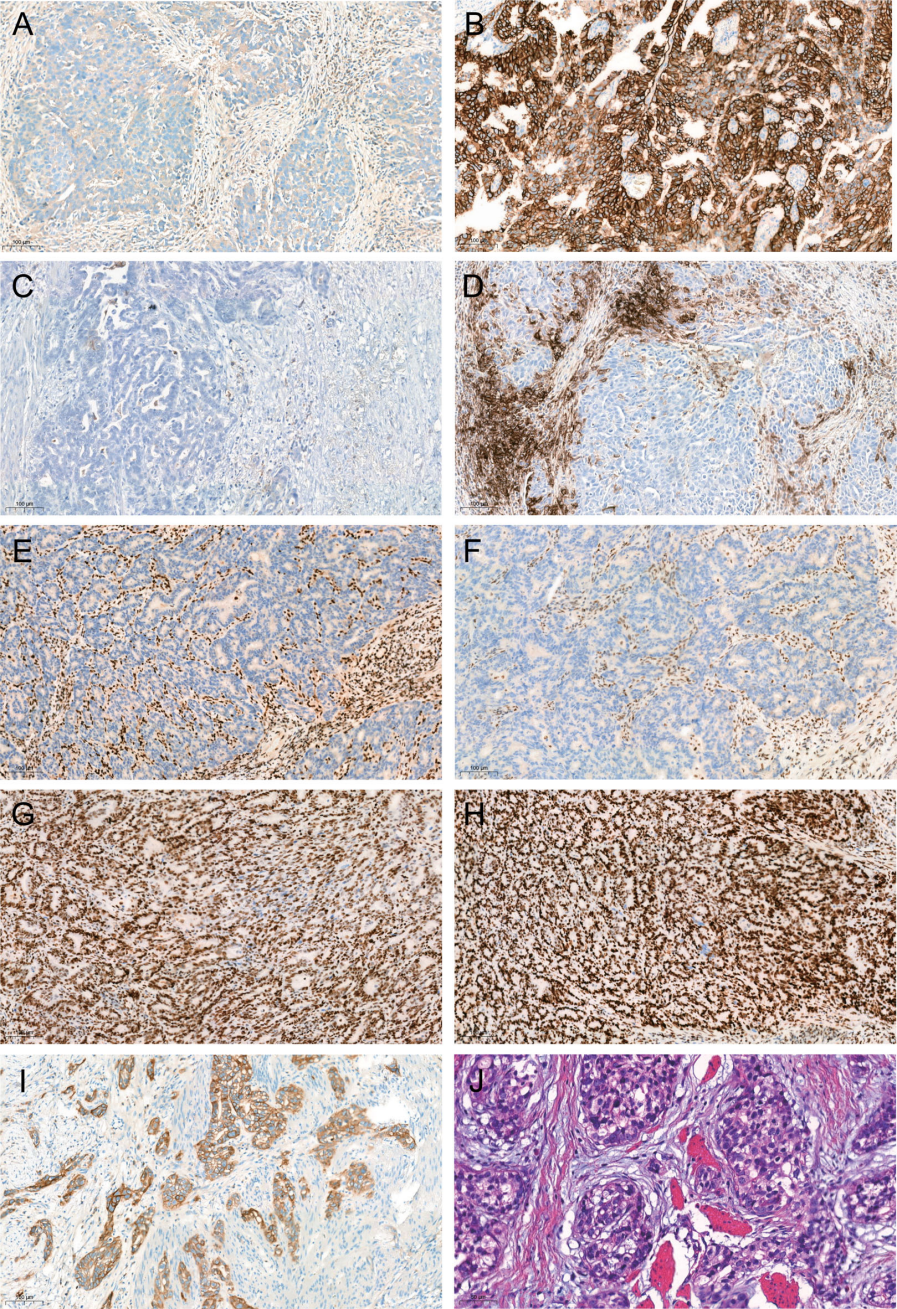
Pathological features. Representative images of typical immunohistochemical and pathological features of (A) HER2 0, (B) HER2 3+, (C) CPS below 1, (D) CPS = 60, absence of (E) MLH1 and (F) PMS2, intact expression of (G) MSH2 and (H) MSH6, (I) cmet 3+, and (J) HE‐stained section of hepatoid adenocarcinoma of stomach.

PD‐L1 antibody clone 22C3 was evaluated using the CPS. In brief, PD‐L1 IHC was conducted to assess cell membrane staining. Additionally, the microsatellite instability (MSI) group was identified by the complete loss of at least one of the four mismatch repair (MMR) proteins (MLH1, PMS2, MSH2, and MSH6). Furthermore, the expression intensity of cmet was evaluated and categorized as strong (score 3+), moderate (score 2+), weak (score 1+), or absent (score 0) (Figure 1).

### Statistical analysis

Continuous variables were compared using the Student's *t* test if normally distributed, otherwise compared by the Mann–Whitney *U* test. Categorical variables were compared by Pearson's chi‐square test or Fisher's exact test determined by the sample size if unordered, otherwise compared by the Mann–Whitney *U* test. Survival analyses were performed by the Kaplan–Meier method. Statistical analyses were conducted using SPSS® (IBM Corp., Armonk, NY, USA, version 26) and relative curves were plotted with OriginPro 2022 (OriginLab Corp., Northampton, MA, USA, version 2022). If *p* < 0.050, the differences were considered as significant statistically.

### Ethics approval and consent to participate

The Ethics Committee of Peking University Cancer Hospital and Institute (code PY202329) approved this study. Informed consent was obtained from all participants.

## Results

### Patient enrollment

**Figure 2:**
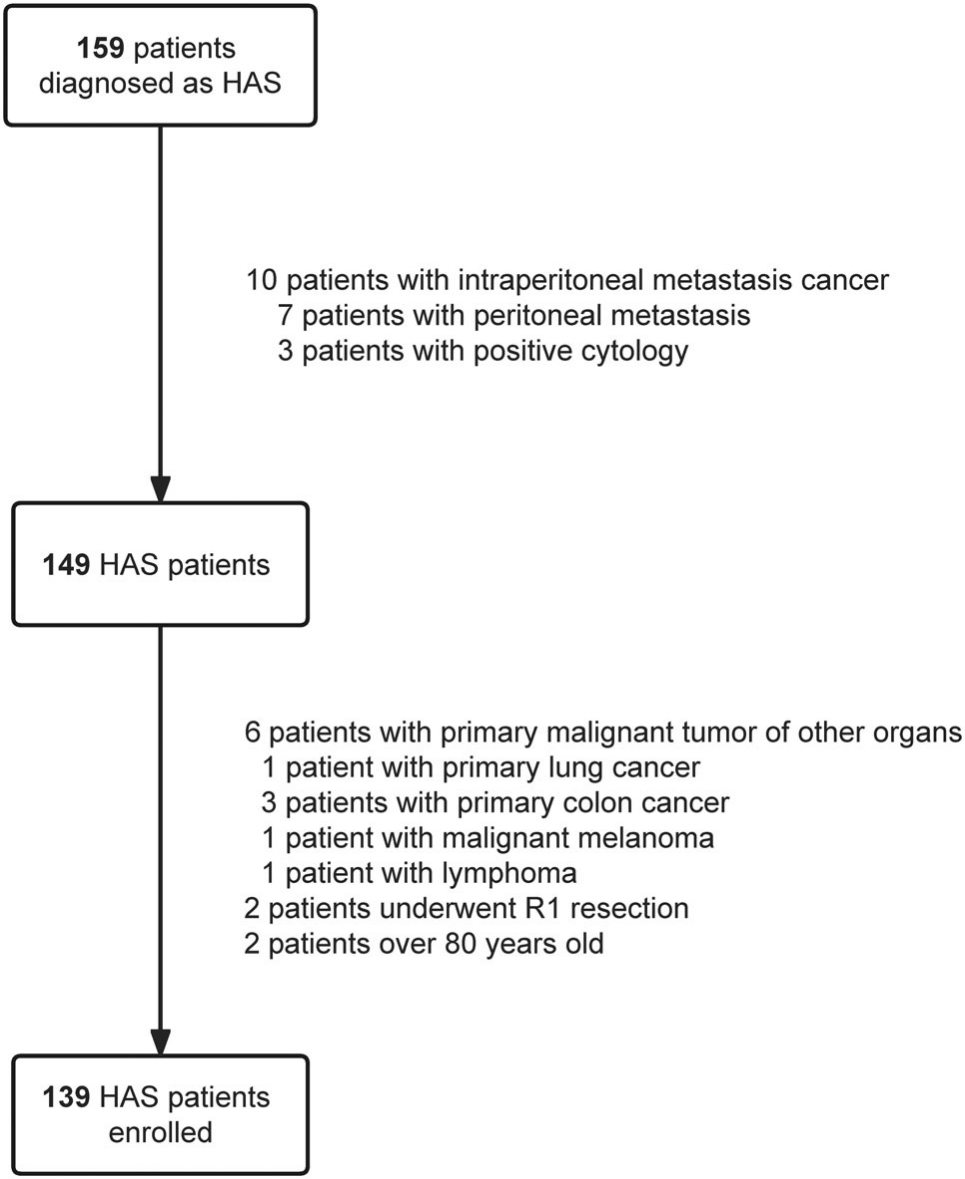
Flow diagram. A total of 159 patients were diagnosed as HAS in our center. Twenty patients were excluded according to the exclusion criteria, leaving 139 patients enrolled in this study.

A total of 159 gastric cancer patients underwent gastrectomy and were diagnosed with HAS at the Center of Gastrointestinal Cancer of Peking University Cancer Hospital from October 2009 to July 2021. According to the exclusion criteria, 20 patients were excluded: 10 patients with intraperitoneal metastasis before the operation (7 patients with peritoneal metastasis and 3 patients with positive cytology), 2 patients who underwent R1 resection, 2 patients over 80 years old, 6 patients with a primary malignant tumor of other organs (1 patient with primary lung cancer, 3 patients with primary colon cancer, 1 patient with malignant melanoma, and 1 patient with lymphoma). Consequently, a total of 139 patients were enrolled in this research (Figure 2).

### EBER and MMR status

**Figure 3:**
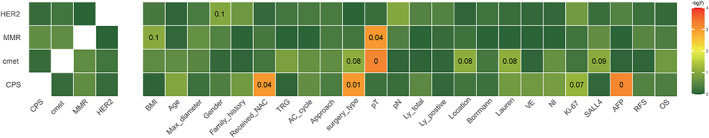
Correlation heatmap of different IHC and clinical traits. Correlation heatmap showing IHC, clinical features, and survival outcomes along the horizontal axis and IHC along the vertical axis. The color depth of each lattice represents the relevance of these intersecting factors, according to the legend in the figure. When the statistical *p* value is below or equal to 0.1, the exact number is marked in the figure. BMI, Age, Max_diameter, AC_cycle, Ly_total, and Ly_positive were analyzed as continuous variables. Gender, Family_history, Received_NAC, TRG, Approach, Surgery_type, pT, PN, Location, Borrmann, Lauren, VE, NI, Ki‐67, SALL4, and AFP were analyzed as categorical variables. AC_cycle, number of adjuvant chemotherapy cycles; AFP, alpha fetoprotein; BMI, body mass index; Ly_positive, number of positive lymph nodes; Ly_total, total number of examined lymph nodes; NAC, neoadjuvant chemotherapy; NI, neural invasion; pN, pathological N stage; pT, pathological T stage; SALL4, spalt like transcription factor 4; TRG, tumor regression grade; VE, vascular embolism.

EBER analysis was performed in 113 of the 139 patients and all were negative. MMR protein IHC for MLH1, MSH2, MSH6, and PMS2 was performed in 138 patients. MMR deficiency (dMMR) was defined as the absence of expression of any of the four MMR proteins and the MMR proficiency (pMMR) was defined as the intact expression of all MMR proteins. dMMR was identified in two (1.4%) patients; both cases showed intact expression of MSH2 and MSH6, and absent expression of MLH1 and PMS2. Analysis of MMR status and other clinicopathological features found that the MMR status was related to tumor stage (Figure 3); both of these dMMR patients were pT4 and pN2. Detailed clinical data are shown in Table 1.

**Table 1:** Detailed clinical data for the two dMMR HAS patients

	Gender	Age/years	pT	pN	VE	NI	Received NAT	TRG	CPS	HER2	AT regimen	OS*	OS time/month	RFS*	RFS time/month
Case 59	Female	62	4	2	No	No	No	–	0	0	SOX Six cycles	0	36	0	36
Case 101	Male	64	4	2	Yes	Yes	Yes Folfox Two cycles	3	0	0	Toripalimab + Bev Six cycles	0	36	1 (liver metastasis)	7

AT, adjuvant therapy; NAT, neoadjuvant therapy; NI, neural invasion; pN, pathological N stage; pT, pathological T stage; TRG, tumor regression grade; VE, vascular embolism.

* In OS and RFS, 1 represents death and 0 represents survival at the follow‐up period.

### AFP and spalt like transcription factor 4 (SALL4)

AFP IHC was performed in 138 of 139 HAS patients and was positive in 128 (92.8%) of these. SALL4 IHC was performed in 128 of 139 HAS patients and was positive in 110 (85.9%) of these. Overall, 138 of 139 HAS patients harbored at least one positive IHC stain for AFP or SALL4 (AFP and SALL4 were not tested in the remaining patient). The relationship between AFP/SALL4 status and other clinical features is illustrated in Figure 3.

### HER2 IHC and FISH

**Figure 4:**
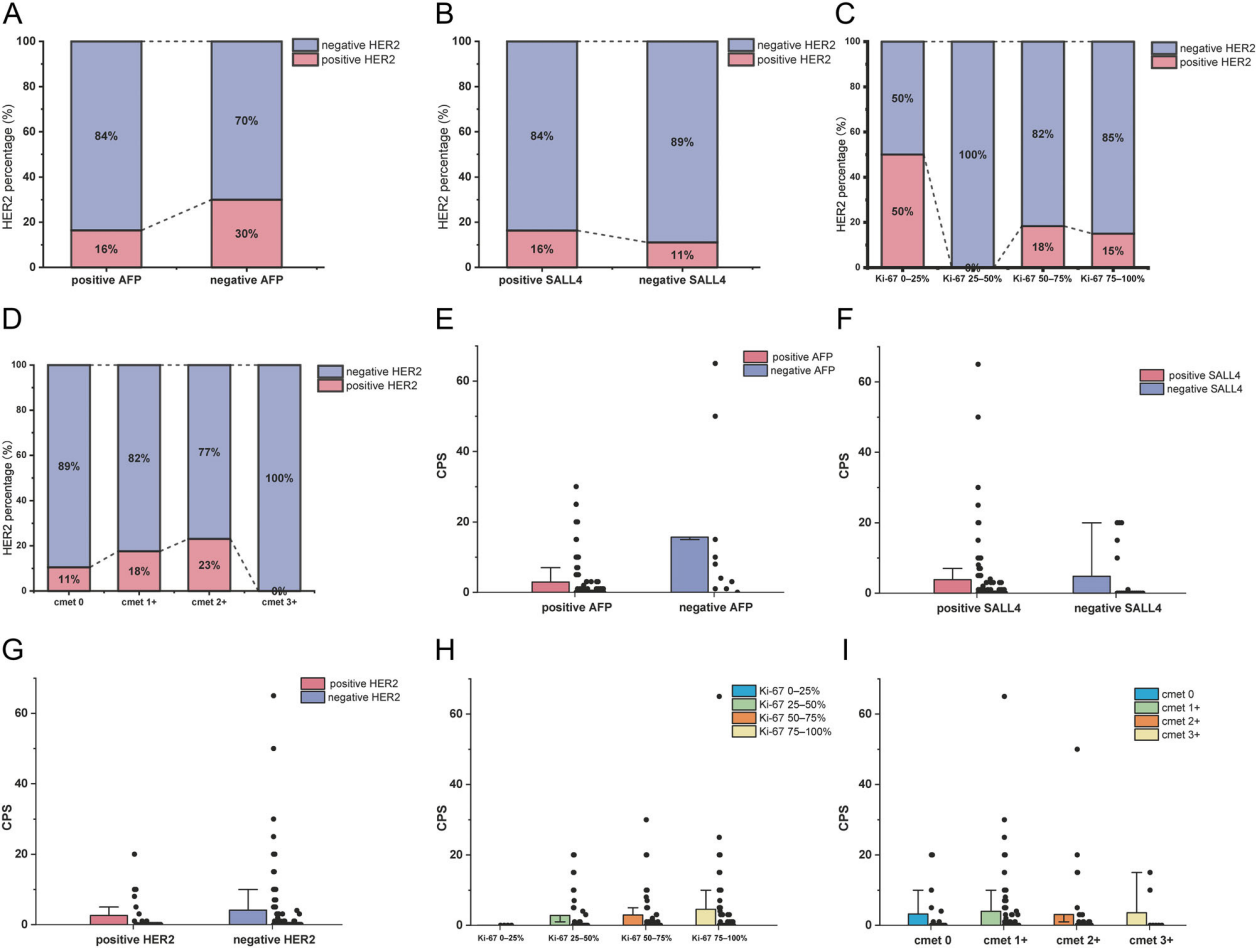
Immunohistochemical findings and correlations. The relationship between HER2 and (A) AFP, (B) SALL4, (C) different Ki‐67 levels, and (D) cmet expression status. All these factors have no statistical significance with HER2 expression. The relationship between CPS and (E) AFP, (F) SALL4, (G) HER2, (H) different Ki‐67 levels, and (I) cmet expression status. HAS patients with a negative AFP stain tend to have a higher CPS, but there is no statistically significant difference. The other IHC parameters have no statistical significance with CPS.

IHC for HER2 was performed in all 139 HAS patients. Among them, 109 patients showed HER2 0 or 1+, 8 patients showed HER2 2+, and 22 showed HER2 3+. We then performed a FISH examination in the eight HER2 2+ patients and two patients presented FISH amplification. Overall, 24 (17.3%) HAS patients had positive HER2 status and 115 (82.7%) HAS patients were negative. Further analyses found that female HAS patients were prone to have positive HER2. Twenty eight percentage of female patients showed positive HER2, while only 15% male patients showed positive HER2. However, statistical difference was not achieved (*p* = 0.10). The detailed relationship between HER2 status and other clinical features is illustrated in Figures 3, 4 and supplementary material, Figure S1.

### PD‐L1 CPS

A total of 138 patients in this study received a CPS review. Among them, 82 (59.4%) patients showed negative CPS (CPS = 0), and 56 (40.6%) patients had a CPS equal to or greater than 1. HAS patients with negative AFP tended to have a higher proportion with positive CPS (CPS > 0). Ninety percentage of (9/10) AFP‐negative patients had a positive CPS while 36.7% (47/128) AFP‐positive patients had a positive CPS. Meanwhile, HAS patients with negative AFP had higher CPS (mean CPS 15.7) compared to those with positive AFP (mean CPS 2.92). In addition, we found that all HAS patients with Ki‐67 expression below 25% had negative CPS (CPS = 0). Nevertheless, statistical difference was not achieved (*p* = 0.07). The detailed relationship between CPS status and other clinical features is illustrated in Figures 3, 4, and supplementary material, Figure S2. It should be noted that CPS in Figure 3 was treated as a categorical variable (CPS = 0 versus CPS ≥ 1).

### cmet status

cmet status was available for all 139 HAS patients in this study. Of these, 19 (13.7%) patients showed cmet 0, 74 (53.2%) showed cmet 1+, 39 (28.1%) showed cmet 2+, and 7 (5.0%) showed cmet 3+. In general, cmet 3+ was defined as overexpression in the cmet IHC stain. Correlation analyses found that cmet expression was related to pT stage. Thus, we further analyzed the relationship between cmet expression status and pT stage and found that cmet overexpression frequently occurred in the pT4 stage; 24% (5/21) HAS patients in the pT4 stage showed cmet overexpression compared with 0% in the pT1 stage, 3% in the pT2 stage, and 1% in the pT3 stage, respectively (supplementary material, Figure S3). The detailed relationship between cmet status and other clinical features is illustrated in Figure 3.

### The relationship between IHC analysis and survival outcomes

**Figure 5:**
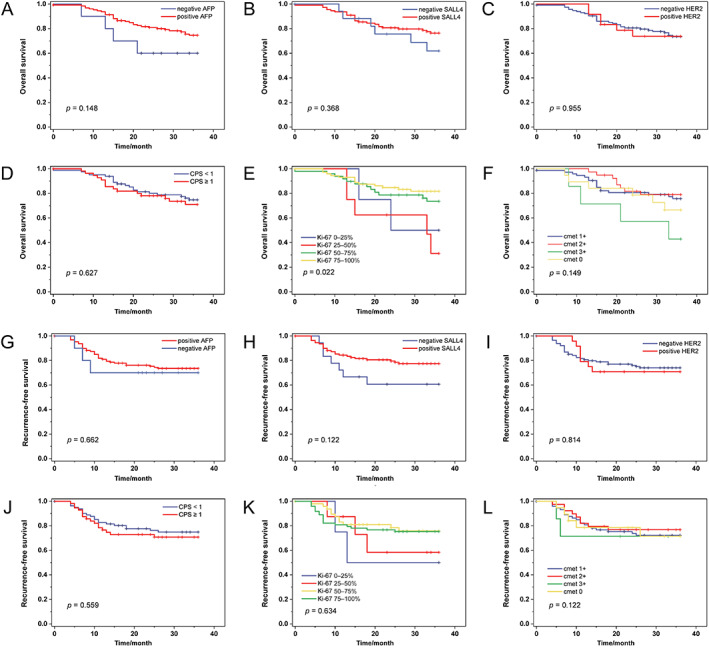
The survival analysis of HAS patients with different IHC status. HAS patients with different (A, G) AFP expression status, (B, H) SALL4 expression status, (C, I) HER2 expression status, (D, J) CPS status, (E, K) Ki‐67 expression status, and (F, L) cmet expression status have no significant difference in OS or RFS outcomes.

We performed survival analyses of different IHC markers, and found they had no statistical relationship with OS and RFS (Figure 5). However, we noticed that both dMMR patients achieved long‐term survival, though both cases were at a locally advanced stage by pathological review and liver metastasis occurred in case 101 in the seventh month after the treatment (Table 1). Nonetheless, there was no statistical difference in survival between dMMR and pMMR patients, which might be due to the small sample size of dMMR patients.

### Emerging therapies and survival

**Figure 6:**
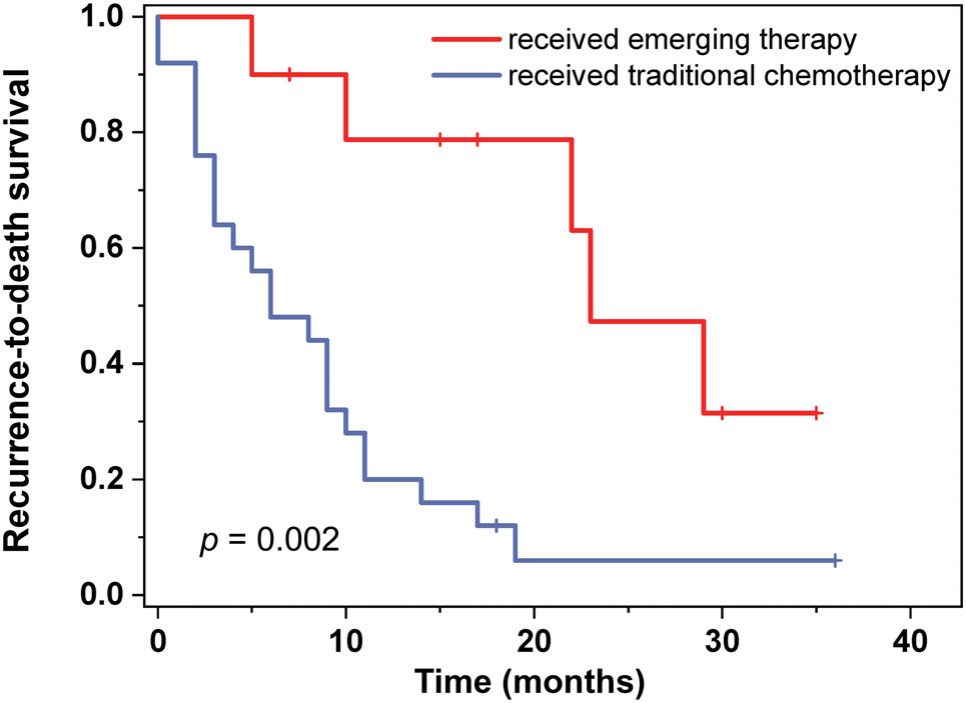
The survival analysis of recurrent HAS patients with different treatments. HAS patients with recurrent disease who received emerging therapy have a significantly better survival outcome than those received traditional chemotherapy.

To explore the effect of immunotherapy and anti‐angiogenesis therapy among HAS patients, we reviewed the survival outcome of recurrent HAS patients. We defined immunotherapy with or without anti‐angiogenesis therapy as emerging therapy and platinum and fluorouracil‐based chemotherapy as traditional chemotherapy. We compared the clinicopathological characteristics of these two groups, and found that there was no significant difference among them (supplementary material, Table S2). Survival analysis showed a significant difference between recurrent HAS patients who received emerging therapy and traditional chemotherapy (*p* = 0.002, median RDS: 23 months versus 6 months). Recurrent HAS patients who received emerging therapy had an obviously better prognosis than those who received traditional chemotherapy (Figure 6).

#### Patients who received anti‐HER2 therapy

Five patients in our study received anti‐HER2 (or Herceptin) therapy. Their clinicopathological data are shown in Table 2. Impressively, all these patients achieved long‐term OS and RFS, despite some harboring poor prognostic indices. For instance, case 96 was a pT3N2 stage tumor that presented with vascular embolism as well as (peri)neural invasion; however, after receiving six cycles of perioperative chemotherapy combined with anti‐HER2 therapy, the patient remained free of recurrence and was still alive at the end of the follow‐up period (Table 2).

**Table 2:** Detailed clinical data for the five HAS patients who received Herceptin therapy

	Gender	Age/years	pT	pN	VE	NI	Received NAC	TRG	CPS	HER2	AC regimen	OS*	OS time/month	RFS*	RFS time/month
Case 41	Male	63	1	1	No	No	Yes Xelox + Herceptin Four cycles	2	0	3+	Herceptin NA cycle	0	36	0	36
Case 54	Male	53	3	1	No	No	Yes Xelox Two cycles	3	0	0	Paclitaxel + Capecitabine + Herceptin NA cycle	0	36	0	36
Case 71	Male	56	3	2	No	Yes	Yes SOX Two cycles	3	1	3+	SOX + Herceptin Three cycles	0	22	0	22
Case 82	Male	67	2	1	Yes	Yes	Yes SOX + Herceptin Two cycles	2	0	1+	SOX + Herceptin Four cycles; S1 Two cycles	0	36	0	36
Case 96	Male	59	3	2	Yes	Yes	Yes POS + Herceptin One cycle	2	20	3+	POS + Herceptin Five cycles	0	36	0	36

AC, adjuvant chemotherapy; NAC, neoadjuvant chemotherapy; NI, neural invasion; pN, pathological N stage; pT, pathological T stage; TRG, tumor regression grade; VE, vascular embolism.

* In OS and RFS, 1 represents death and 0 represents survival at the follow‐up period.

## Discussion

This is a retrospective single‐center study exploring IHC and potential therapeutic approaches of HAS with a relatively adequate sample size. Proposed in 1985, HAS was defined as primary gastric cancer with elevated serum AFP and hepatoid areas in a pathological review by Ishikura *et al* [24]. However, the diagnostic criteria have been debated. In 1993, Nagai *et al* found that HAS had a poor prognosis regardless of whether it produced AFP, and they suggested that the morphologic pathological appearance should be essential for diagnosing HAS [25]. Nagai *et al*'s diagnostic criteria were acknowledged by an increasing number of researchers, and also accepted by the WHO diagnostic criteria. According to the fifth edition WHO classification of digestive system tumors [6], HAS was identified as an independent subtype of gastric cancer relying on its unique pathological features. However, some researchers argued that WHO criteria might lead to incorrect or missed diagnosis of HAS. For example, Xie *et al* found that 30.8% (8/26) of HAS and AFP‐producing gastric cancer (AFPGC) patients were incorrectly diagnosed [7]. Similarly, Kong *et al* found that 31 of 34 HAS patients had been misdiagnosed as common adenocarcinoma, with a 91.2% misdiagnosis rate [5]. On the other hand, we found that nearly all HAS patients exhibited IHC positivity for AFP or SALL4. These results agree with previous studies. In 2019, Tsuruta *et al* found that 40 of 52 HAS expressed at least one of the following by IHC: AFP, GPC‐3, SALL4, HepPar‐1, and arginase [26]. Similarly, Kong *et al* found that 73.5% of HAS were AFP positive by IHC in 2021 [5]. Thus, we thought that these distinctive IHC features should be fully utilized to improve the accuracy of HAS diagnosis.

In this study, we found that almost all HAS were MSS and EBER negative. As is well known, gastric cancer was categorized into four subtypes according to molecular features by the Cancer Genome Atlas (TCGA) [27, 28, 29]: EBV‐positive subtype with positive EBER; MSI subtype with complete loss of at least one of four MMR protein (MLH1, MSH2, PMS2, and MSH6); genomically stable (GS) subtype with frequent E‐cadherin gene 1 (*CDH1*) mutation; and chromosome instability (CIN) subtype with frequent *TP53* mutation. According to the TCGA classification, most HAS patients in our study likely belong to the GS or CIN subtype. Similarly, in 2021, He *et al* analyzed 55 HAS patients and found that all these patients belonged to the CIN/GS group [16]. In 2022, Kang *et al* enrolled 36 HAS patients and also found that all patients belonged to CIN/GS [11]. Interestingly, they all found frequent mutations in the *TP53* gene. Given that the CIN subtype features *TP53* gene mutation, we speculated that the majority of HAS might belong to the CIN subtype. Recently, the Asian Cancer Research Group (ACRG) proposed a new molecular classification on the basis of Asian populations (mainly from Japan and Korea) [30, 31]: MSI, MSS/*TP53*+ (which means intact *TP53*), MSS/*TP53*−, and MSS/EMT (epithelial to mesenchymal transition). ACRG classification might be more suitable for HAS molecular typing since HAS has mainly been reported in East Asian countries. According to the ACRG classification, HAS mainly belongs to MSS/*TP53*−, which also partly accounts for its poor prognosis compared with common adenocarcinoma of gastric cancer. In our study, we found two HAS patients with dMMR. Likewise, Tsuruta *et al* also found three HAS cases with MSI in 2019 and they argued that few HAS with MSI existed [26]. Interestingly, both HAS patients with dMMR in our study exhibited long‐term survival, though they all had a relatively higher pathological stage, and one of them even developed liver metastasis in the seventh month after the treatment. As a rare subtype of HAS, we knew little about it. However, we suggest that comparatively aggressive treatments for these patients might be a viable option for a better prognosis.

The HAS patients in our research showed a high proportion of positive CPS. This result indicated that HAS patients might benefit from immunotherapy. Subsequent survival analysis supported this hypothesis: the recurrent HAS patients who received immunotherapy with or without anti‐angiogenesis therapy achieved more favorable survival than those with traditional platinum and fluorouracil‐based chemotherapy. Kang *et al* enrolled 36 HAS patients in 2022 and found 9 (25%) patients with positive PD‐L1 (CPS = 1) [11]. However, no patients in their study had higher scores. They also found that some HAS patients exhibited higher tumor‐infiltrating lymphocytes. This result suggested that some HAS patients might have a good immunotherapy response, though almost all HAS patients belonged to CIN or GS which have been considered to have low immunotherapy response rates. In 2021, He *et al* performed IHC for CD3/CD8 and used immunoscore to grade the immune cell infiltration [16]. They found that immunoscore was an independent risk factor for OS and they thought immunotherapy was a promising treatment method for HAS patients. In 2022, Zhao *et al* found that 10.53% of HAS patients harbored tumor mutation burden (TMB) >10 muts/Mb [32]. Likewise, Jiang *et al* found HAS patients harbored more TMB than non‐HAS patients (mean TMB: 18.5/Mb versus 12.6/Mb, *p* = 0.021) [13]. As is well known, patients with high TMB levels, in general, have a better response to immunotherapy. More impressively, Liu *et al* performed single‐cell RNA analysis for a HAS case in 2021 and they revealed remarkable heterogeneity of CD8^+^ T‐cell functional states [18]. The expression of both the activation markers (GZMA and IFNG) and exhaustion markers (PDCD1 and CTLA4) formed a distinctive immune microenvironment. These new tumor antigens could also partly account for the favorable effect of immunotherapy in HAS. Li *et al* enrolled 21 advanced HAS and AFPGC patients in 2020 and they reached similar conclusions [12]. Patients who received immunotherapy or anti‐angiogenesis in their study also showed a better progression free rate and OS rate statistically.

We also found that 17.3% HAS patients in our study had positive HER2 by IHC and FISH. This was also in accord with previous studies. For example, Akazawa *et al* found that 37.5% AFPGC patients had *ERBB2* amplification by next generation sequencing (NGS) and 22% HAS patients were HER2 positive (+++ by IHC or amplification by FISH) [33]. Similarly, He *et al* found 21.8% HAS patients showed positive HER2 (+++ by IHC or amplification by FISH) [16] and Tsuruta *et al* found 21% HAS patients showed HER2 +++ and 19% HAS patients showed HER2 ++ by IHC [26]. In 2022, Jiang *et al* investigated somatic copy number variation (CNV) landscapes by NGS and they found HAS patients harbored more 17q12 amplification than non‐HAS patients (*p* = 0.044) [13]. Of note, ERBB2, together with STARD3 and CDK12, is located on 17q12. These results indicate that HAS patients might benefit from anti‐HER2 therapy. However, a relevant clinical study is rare to date. To our knowledge, the five HAS patients who received anti‐HER2 therapy in our studies are the largest series. All these patients achieved long‐term survival, and we argue that anti‐HER2 therapy is a promising method for HAS patients. Considering the aggressive features and limited treatment modalities for HAS, adjuvant therapy with standard chemotherapy plus anti‐HER2 drugs may be a promising strategy to improve the survival of HAS patients. Further research should be encouraged to explore their effects.

In this research, we found that cmet protein was positive in 86.3% HAS patients and overexpressed in 5% HAS patients. In particular, HAS patients in the pT4 stage harbored a higher proportion of cmet overexpression. Liu *et al* performed WES in 54 HAS patients and found 33 (61%) patients harbored *MET* gene CNV [18]. Thirty‐one patients among them had *MET* gene amplification and five patients had a copy number over 5. *MET* gene was a potential therapeutic target in a lot of cancers, especially in NSCLC [20]. It has been suggested that activation of the *MET*/HGFR pathway is a primary oncogenic driver in NSCLC and an important acquired resistance mechanism in response to first‐line osimertinib treatment [21]. There are four main mechanisms that could result in aberrant activation of the *MET* gene, including gene amplification, overexpression, structural rearrangements, and *MET* exon 14 skipping mutation [19]. It has been acknowledged that *MET* exon 14 skipping mutation is an oncogenic targetable driver mutation in NSCLC [19]. And *MET* inhibitors, like crizotinib and capmatinib, have been widely used in NSCLC patients with positive *MET* and undergoing progression after osimertinib treatment. Interestingly, NSCLC patients with wild‐type *EGFR* gene, amplification of *MET* gene, and overexpression of cmet protein achieved higher objective remission rate after undergoing *MET* inhibitor therapy [21]. And in the sequencing research of HAS, Liu *et al* found that almost all HAS patients had wild‐type *EGFR* (53/54, 98%) [18]. In addition, it has been reported that the rate of *MET* amplification and *MET* exon 14 alterations are about 1.0–5.6% and 1.7–4.3% in NSCLC [20], which is apparently lower than in HAS. Nonetheless, targeted therapy against *MET* has yielded promising results in NSCLC and therefore we suspect that anti‐*MET* therapy might achieve the preferred effect among HAS patients.

The different IHC results in our study did not show statistically significant relationships with the survival outcome of HAS. We thought there were two main reasons. First, all HAS patients in our study were in the locally advanced stage and had undergone radical gastrectomy. And residual circulating tumor cells might resolve after traditional chemotherapy. For HAS patients at this stage, radical gastrectomy and traditional chemotherapy have already achieved satisfactory effects. Second, most of the patients with therapeutic targets in our study received traditional chemotherapy, instead of targeted therapy, after radical gastrectomy. And the improvement in targeted therapy to survival outcome was concealed. On the contrary, a better prognosis had been achieved by emerging therapy in recurrent HAS patients.

There are still several limitations in our study. First, this is a retrospective study in the real world. Due to the rarity of HAS, selection bias and mismatching in clinical data are inevitable. Second, all HAS patients in our study were at a locally advanced stage. Most patients at this stage could achieve long‐term survival after radical surgery and adequate systemic chemotherapy. And the effect of targeted therapy would be concealed to some extent. In summary, prospective research is still needed to validate our conclusion.

## Conclusion

Almost all HAS are MMR proficient, EBER negative, and positive for AFP/SALL4; and a high proportion are HER2 positive and have a positive CPS. Survival analyses indicate that patients with recurrent HAS treated by anti‐HER2 and emerging therapies have better survival than those treated by traditional chemotherapy. In addition, targeted *MET* gene therapy may achieve the preferred effect.

## Author contributions statement

XY, YW and AW designed the study. XY performed statistical analyses and wrote the original manuscripts. YW and XM performed pathological review and IHC experiments. AW helped with figure illustration and revised the manuscript. KZ and KJ assisted with information collection. XJ, JZ and XW provided professional consultancy. ZL and ZB supervised the project. All authors have read and approved the final manuscript.

## Supporting information


**Figure S1.** The relationship between HER2 and other clinical features
**Figure S2.** The relationship between CPS and other clinical features
**Figure S3.** The relationship between cmet expression status and pT stage
**Table S1.** Details of the primary antibodies used in this study
**Table S2.** The clinicopathological features of recurrent HAS patients

## Data Availability

The datasets analyzed during the current study are not publicly available, but are available from the corresponding author on reasonable request.
