# Supplementary material for: Immunohistochemical characteristics and potential therapeutic regimens of hepatoid adenocarcinoma of the stomach: a study of 139 cases
Source: J Pathol Clin Res. 2023 Nov 16;10(1):e343. doi: 10.1002/cjp2.343 (PMC10766033; doi:10.1002/cjp2.343)
Supplement: Supplementary file 1 — Figure S1. The relationship between HER2 and other clinical features Figure S2. The relationship between CPS and other clinical features Figure S3. The relationship between cmet expression status and pT stage Table S1. Details of the primary antibodies used in this study Table S2. The clinicopathological features of recurrent HAS patients [file CJP2-10-e343-s001.pdf]

Immunohistochemical characteristics and potential therapeutic regimens of hepatoid adenocarcinoma of the stomach: a study of 139 cases

X Yang, Y Wu, A Wang *et al*., *J Pathol Clin Res*, https://doi.org/10.1002/cjp2.343

Supplementary Figures S1–S3

Supplementary Tables S1 and S2

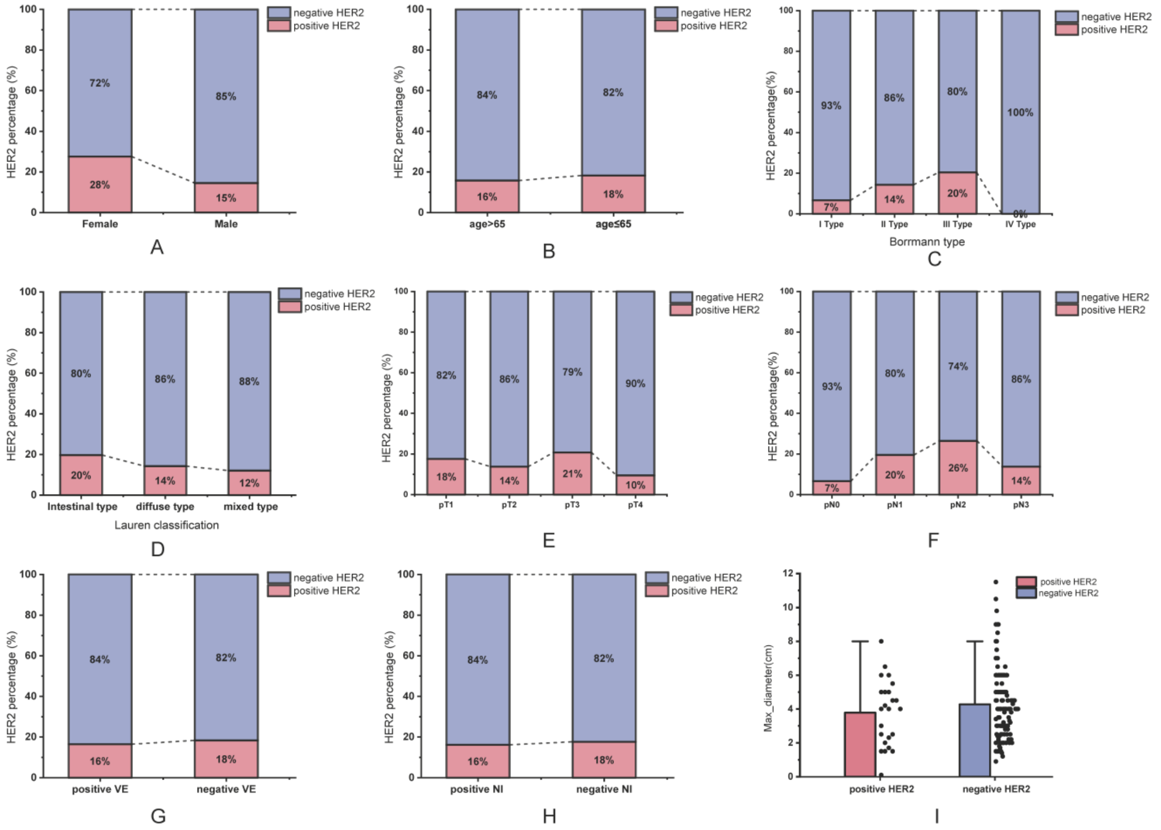

Figure S1. The relationship between HER2 and other clinical features.

The relationship of HER2 and (A) gender, (B) age, (C) Borrmann type, (D) Lauren classification, (E) pT stage, (F) pN stage, (G) VE, (H) NI and (I) maximum tumor diameter. All these factors have no statistical significance with HER2 expression. VE, vascular embolism; NI, neural invasion.

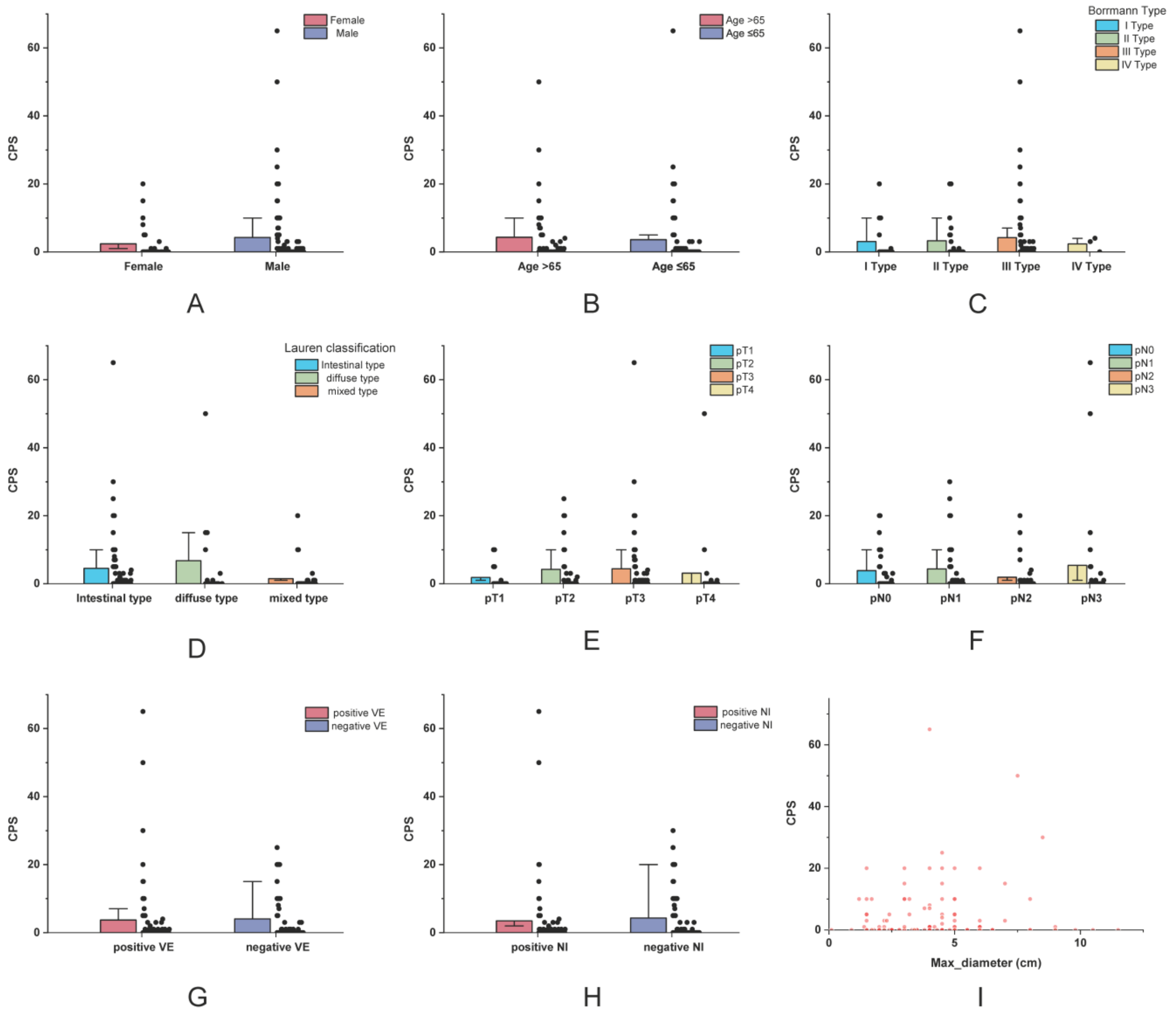

Figure S2. The relationship between CPS and other clinical features.

The relationship of CPS and (A) gender, (B) age, (C) Borrmann type, (D) Lauren classification, (E) pT stage, (F) pN stage, (G) VE, (H) NI and (I) maximum tumor diameter. All these factors have no statistical significance with CPS. VE, vascular embolism; NI, neural invasion.

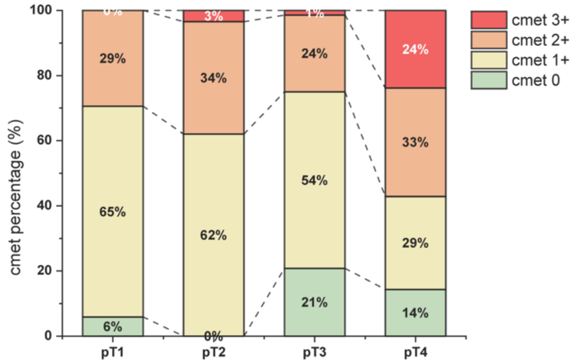

Figure S3. The relationship between cmet expression status and pT stage. HAS patients with pT4 stage have a higher proportion of cmet 3+.

Table S1. Details of the primary antibodies used in this study

Primary antibodies	Company	IHC platforms
EGFR	Zhongshan Golden Bridge Biotechnology, Beijing, PR China	UltraPATH fully automated IHC staining system
HER-2 (4B5)	Roche Diagnostics, Risch-Rotkreuz, Switzerland	Roche Benchmark XT automated staining system
Cmet	Roche Diagnostics, Risch-Rotkreuz, Switzerland	Roche Benchmark XT automated staining system
PD-L1 (22C3)	Dako, Carpinteria, CA, USA	Dako PD-L1 IHC 22C3 pharmDx
MMR proteins	Gene Tech, Shanghai, PR China	Leica Bond III automated platform
AFP	Zhongshan Golden Bridge Biotechnology, Beijing, PR China	Leica Bond III automated platform
SALL4	Zhongshan Golden Bridge Biotechnology, Beijing, PR China	Leica Bond III automated platform

Table S2. The clinicopathological features of recurrent HAS patients

Variables	Total (*n* = 36)	Traditional therapy (*n* = 25)	Emerging therapy (*n* = 11)	*p*
**c-met***				0.614
0	5 (14)	4 (16)	1 (9)	
1	20 (56)	12 (48)	8 (73)	
2	9 (25)	7 (28)	2 (18)	
3	2 (6)	2 (8)	0 (0)	
**MMR***				0.306
pMMR	35 (97)	25 (100)	10 (91)	
dMMR	1 (3)	0 (0)	1 (9)	
**CPS***				0.159
0	20 (56)	16 (64)	4 (36)	
≥1	16 (44)	9 (36)	7 (64)	
**HER2_status***				0.4
Negative	29 (81)	19 (76)	10 (91)	
Positive	7 (19)	6 (24)	1 (9)	
**Gender***				1
Female	6 (17)	4 (16)	2 (18)	
Male	30 (83)	21 (84)	9 (82)	
**Age#**	60.47 ± 11.1	61.52 ± 11.2	58.09 ± 11.02	0.403
**BMI†**	24.2 (23.17, 25.95)	24.09 (23.39, 25.47)	25.26 (22.97, 27.68)	0.481
**Family_history***				0.65
Without	29 (81)	21 (84)	8 (73)	
With	7 (19)	4 (16)	3 (27)	
**Received_NAC***				0.825
No	19 (53)	14 (56)	5 (45)	
Yes	17 (47)	11 (44)	6 (55)	
**TRG***				0.11
1	2 (12)	0 (0)	2 (33)	
2	1 (6)	1 (9)	0 (0)	
3	14 (82)	10 (91)	4 (67)	

**Surgical approach**[*]				0.552
Open	27 (75)	18 (72)	9 (82)	
Lap.	7 (19)	6 (24)	1 (9)	
Multivisceral resection	2 (6)	1 (4)	1 (9)	
**Surgery_type**[*]				0.741
Proximal	2 (6)	1 (4)	1 (9)	
Distal	15 (42)	10 (40)	5 (45)	
Total	19 (53)	14 (56)	5 (45)	
**pT**[*]				0.939
1	2 (6)	1 (4)	1 (9)	
2	6 (17)	4 (16)	2 (18)	
3	21 (58)	15 (60)	6 (55)	
4	7 (19)	5 (20)	2 (18)	
**pN**[*]				0.227
0	4 (11)	3 (12)	1 (9)	
1	10 (28)	5 (20)	5 (45)	
2	13 (36)	11 (44)	2 (18)	
3	8 (22)	6 (24)	2 (18)	
4	1 (3)	0 (0)	1 (9)	
**Ly_total**[#]	32.22 ± 12.86	29.12 ± 10.23	39.27 ± 15.79	0.071
**Ly_positive**[†]	5 (1, 7)	5 (2, 7)	2 (1, 7.5)	0.756
**Location**[*]				0.529
Upper	17 (47)	11 (44)	6 (55)	
Middle (or unclear)	4 (11)	4 (16)	0 (0)	
Lower	15 (42)	10 (40)	5 (45)	
**Borrmann**[*]				0.408
1	6 (17)	3 (12)	3 (27)	
2	2 (6)	2 (8)	0 (0)	
3	26 (72)	19 (76)	7 (64)	
4	2 (6)	1 (4)	1 (9)	
**Lauren**[*]				0.053
Intestinal	21 (60)	12 (50)	9 (82)	

Diffuse	5 (14)	3 (12)	2 (18)	
Mixed	9 (26)	9 (38)	0 (0)	
**Max_diameter**#	4.78 ± 2.4	4.39 ± 2.02	5.66 ± 3	0.22
**Vascular.embolism***				1
Without	12 (33)	8 (32)	4 (36)	
With	24 (67)	17 (68)	7 (64)	
**Neural.invasion***				0.708
Without	13 (37)	8 (33)	5 (45)	
With	22 (63)	16 (67)	6 (55)	
**Ki_67***				1
0	2 (6)	2 (9)	0 (0)	
1	3 (9)	2 (9)	1 (9)	
2	11 (32)	7 (30)	4 (36)	
3	18 (53)	12 (52)	6 (55)	
**SALL4***				0.652
Negative	7 (23)	4 (19)	3 (30)	
Positive	24 (77)	17 (81)	7 (70)	
**AFP***				0.538
Negative	3 (8)	3 (12)	0 (0)	
Positive	33 (92)	22 (88)	11 (100)	
**CEA**†	2.95 (1.48, 9.79)	2.85 (1.55, 8.91)	3.71 (1.79, 8.91)	0.807
**CA199**†	11.17 (7.01, 21.17)	11.5 (7.67, 21)	9.6 (6.38, 26.91)	0.693
**CA242**†	4.7 (3.2, 5.54)	4.7 (3.45, 8.87)	4.45 (3.05, 5.47)	0.808
**CA724**†	2.58 (1.54, 4.6)	2.33 (1.44, 4.05)	2.88 (1.89, 7.88)	0.222
**CA125**†	10.76 (8.75, 14.51)	10.42 (8.68, 14.41)	11.43 (9.85, 14.7)	0.506
**AFP**†	16.38 (3.85, 283.82)	16.74 (4.12, 228.75)	4.58 (2.58, 403.35)	0.735

* These parameters are presented as *n* (%).

# These parameters are presented as Mean ± SD.

† These parameters are presented as Median (Q1, Q3).
